# Rifampicin Resistant Tuberculosis in Lesotho: Diagnosis, Treatment Initiation and Outcomes

**DOI:** 10.1038/s41598-020-58690-4

**Published:** 2020-02-05

**Authors:** Bulemba Katende, Tonya M. Esterhuizen, Anzaan Dippenaar, Robin M. Warren

**Affiliations:** 1 Division of Epidemiology and Biostatistics, Stellenbosch University, Tygerberg, South Africa; 2 Elizabeth Glaser Pediatric Aids Foundation, Maseru, Lesotho; 3 DST-NRF Centre for Excellence for Biomedical Tuberculosis Research, SAMRC Centre for Tuberculosis Research, Division of Molecular Biology and Human Genetics, Faculty of Medicine and Health Sciences, Stellenbosch University, Tygerberg, South Africa

**Keywords:** Infectious-disease diagnostics, Molecular medicine

## Abstract

The Lesotho guidelines for the management of drug-resistant tuberculosis (TB) recommend initiation of patients diagnosed with rifampicin resistant (RR)-TB on a standardized drug resistant regimen while awaiting confirmation of rifampicin resistant TB (RR-TB) and complete drug susceptibility test results. Review of diagnostic records between 2014 and 2016 identified 518 patients with RR-TB. Only 314 (60.6%) patients could be linked to treatment records at the Lesotho MDR hospital. The median delay in treatment initiation from the availability of Xpert MTB/RIF assay result was 12 days (IQR 7–19). Only 32% (101) of patients had a documented first-line drug resistant test. MDR-TB was detected in 56.4% of patients while 33.7% of patients had rifampicin mono-resistance. Only 7.4% of patients assessed for second-line resistance had a positive result (resistance to fluoroquinolone). Treatment success was 69.8%, death rate was 28.8%, loss to follow up was 1.0%, and 0.4% failed treatment. Death was associated with positive or unavailable sputum smear at the end of first month of treatment (Fisher exact p < 0.001) and older age (p = 0.007). Urgent attention needs to be given to link patients with RR-TB to care worldwide. The association of death rate with positive sputum smear at the end of the first month of treatment should trigger early individualization of treatment.

## Introduction

In 2018, the World Health Organization (WHO) estimated the number of rifampicin resistant tuberculosis (RR-TB) cases to be 484 000, which is a slight decrease compared to the 558 000 cases estimated globally in 2017^1^. In total, 30 high burden drug resistant TB countries have been identified by the WHO, of which eight are in Africa. In Lesotho (one of the high burden TB countries), 4.8% of new TB cases and 14% of previously treated cases are estimated to have drug-resistant TB^1^. The Lesotho National Tuberculosis program reported 152 cases of drug resistant TB in 2014, 209 cases in 2015 and 245 cases in 2016. More than two thirds of these patients were new cases and HIV positive, while approximately 30% had received prior TB treatment (retreatment cases)^2,3^.

To address the increasing drug-resistant TB epidemic, the Lesotho National Tuberculosis program recommends the use of the Xpert MTB/RIF assay as the first line test for the diagnosis of pulmonary TB. Since 2015, all the government and Christian Health Association of Lesotho’s hospitals were capacitated to provide diagnosis using the Xpert MTB/RIF assay. The implementation of GeneXpert has aimed to improve TB case detection due to the high accuracy of the test, reduce the time to TB diagnosis and treatment initiation due to the rapidity of the test, and improve TB treatment outcomes by earlier identification of patients with RR-TB^4,5^.

The current Lesotho National Guidelines for Tuberculosis recommends initiation of all patients diagnosed with RR-TB using the Xpert MTB/RIF assay on a standard drug resistant TB treatment regimen. This standard regimen may be adjusted with each patient receiving a personal regimen (individualization) based on subsequent drug susceptibility test (DST) results^6^.

Despite the implementation of the GeneXpert in Lesotho, Tuberculosis case detection remains low (48%)^7^. Furthermore, the impact of the Xpert MTB/RIF assay on initiation of drug resistant TB treatment remains unknown. Studies from South Africa reported substantial delays for treatment initiation and high rates of suboptimal treatment for patients diagnosed with RR-TB when initiating treatment based solely on the Xpert MTB/RIF result^8–10^.

In this study, we aimed to determine the proportion of patients with RR-TB linked to care, the turnaround time for treatment initiation, factors associated with delays, and to assess treatment outcomes for patients diagnosed with RR-TB using the Xpert MTB/RIF assay.

## Results

### Participant characteristics and RR-TB distribution

**Figure 1:**
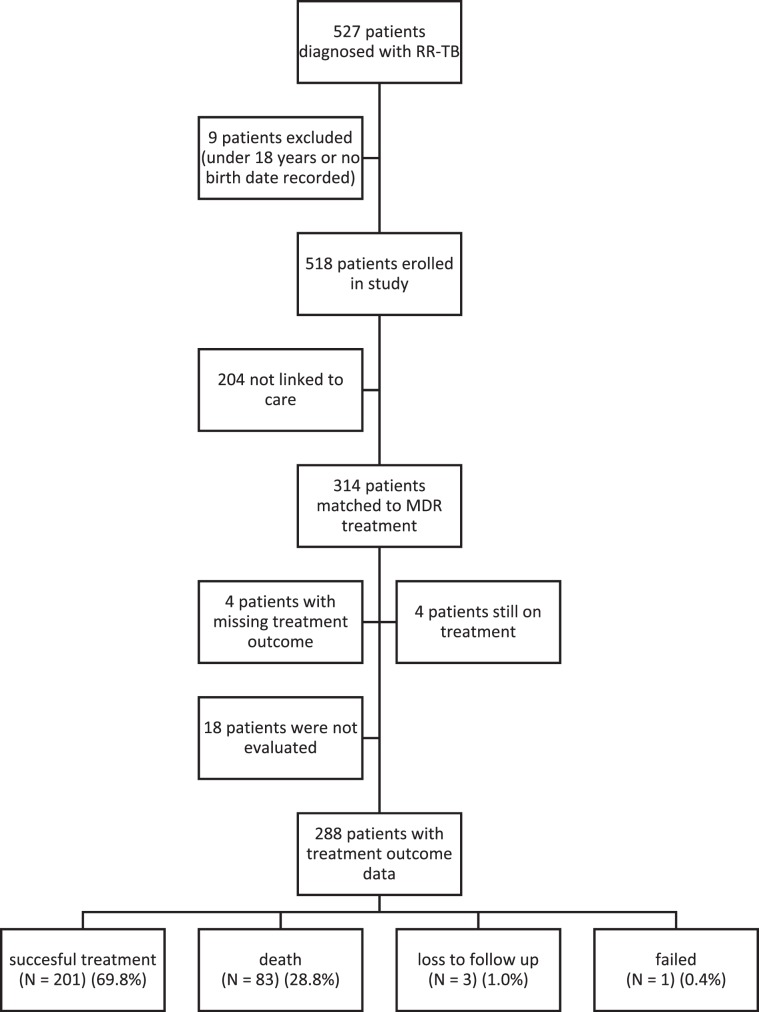
Flow diagram of patients with RR-TB included in the study.

We visited 19 GeneXpert facilities throughout Lesotho and were able to identify 527 patients diagnosed with RR-TB between January 2014 and December 2016 using the Xpert MTB/RIF assay (Fig. 1 (see Supplemental Data)). Nine patients were excluded because they were either under the age of 18 years or had missing dates of birth.

Of the 518 patients diagnosed with RR-TB, 314 (60.6%) were successfully matched to Lesotho MDR Hospital records (Table 1). The remaining 204 (39.4%) patients could not be matched. The mean age between matched and unmatched patients were similar (mean difference 0 .79, 95% CI (1.57–3.15)), there were no differences in gender distribution (X^2^ = 1.51, p = 0.22) or patient distribution per district (X^2^ = 11.70 p = 0.23) between the two groups.

**Table 1:** Primary characteristics of study participants*.

Category		Number of participants (%)
Age group (N = 314)	18–30 years	75 (23.9)
	31–40 years	103 (32.8)
	41–50 years	52 (16.6)
	>50	84 (26.8)
Gender (N = 314)	Male	191 (60.8)
	Female	123(39.2)
Occupation (N = 314)	Mine and Ex-mine workers	59 (19.0)
	Other occupations	105 (33.8)
	No occupation	120 (38.6)
	Unknown occupation	27 (8.7)
	Missing	4 (1.3)
History of previous TB (N = 314)	Yes	134 (42.9)
	No	179 (57.0)
	Unknown	1 (0.3)
HIV status (N = 314)	Positive	245 (78.0)
	Negative	69 (22.0)
Use of ARVs (N = 245)	Yes	201 (82.0)
	No	44 (18.0)
Weight before treatment (kg) (N = 194) Mean (**SD)	53.0 (10.3)	95% ^***^CI 51.5–54.5
Weight after treatment (kg) (N = 194) Mean (SD)	60.1 (11.6)	95% CI 58.5–61.8

*For age and gender N represent the total number of patients included in the study (314), for occupation, history of TB and HIV status N is the number of patients matched at the hospital (314), for use of ARVs N is equal to the number of HIV+ patients (245). For weight N is equal to the number of patients with both pre- and post-treatment weight and with treatment outcome different from active and not evaluated.

^**^Standard deviation.

^***^Confidence interval.

Table 1 gives the primary characteristics of all the study participants. Fifty-seven percent were new cases, 78% were HIV positive and 82% of those with an HIV positive status were already on antiretroviral treatment at the time of treatment initiation. Sixty-one percent were male with most patients being between 31 to 40 years (32.8%). Mean weight before treatment was 53.0kg (95% CI 51.5–54.5) and after treatment 60.1 kg (95% CI 58.5–61.8). A history of working in a South African mine was the most common occupation (19%).

### Treatment delay and associated factors

**Figure 2:**
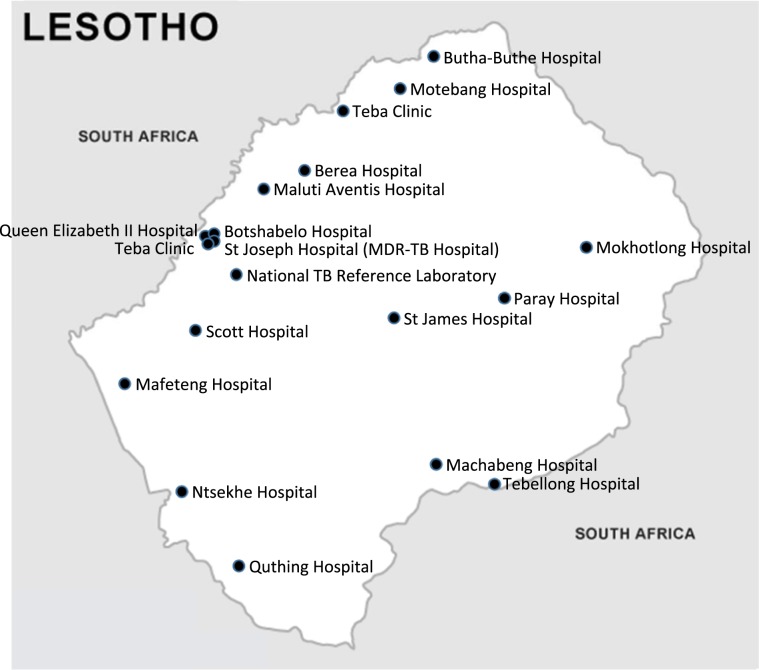
Map of Lesotho showing the location of the Gene Xpert facilities in relation to the MDR-TB hospital.

Of the 314 study patients with matched hospital records, 302 (96.1%) were eligible for analysis. We excluded 12 patients with missing date of treatment initiation. The overall median time to treatment initiation from the availability of GeneXpert results was 12 days (IQR 7–19) (Table 2). Patients from Mokhotlong district had the shortest delay in treatment initiation, 6.5 days (IQR 5–8) while patients from Thaba Tseka had the longest delay, 60 days (IQR 22–70) (Fig. 2). There were no associations between time to treatment initiation and gender (Mann-Whitney, p = 0.94), age group (Kruskal Wallis, X2 = 2.39, 4 d.f., p = 0.66), HIV status (Mann-Whitney, p = 0.81), history of previous TB (Kruskal Wallis, X2 = 0.97, 2 d.f., p = 0.62), or distance between GeneXpert facility and MDR hospital (Spearman’s rho = −0.14, p = 0.02).

**Table 2:** Distributions of patients diagnosed with RR-TB and median delay in initiation of MDR-TB treatment.

Districts	Total (N = 518) (%)	Total attending MDR-TB hospital (N = 314) (Matched) (%)	Gender		*Distance to MDR hospital (km)	Median delay in Initiation of treatment (N = 302, including negative delays) Days (IQ range)
			Male (N = 191)	Female (N = 123)		
Berea	70 (13.5)	44 (63.0)	19	25	58.0	12 (8–18)
Butha Buthe	44 (8.5)	26 (59.1)	19	7	123.0	11 (7–14)
Leribe	82 (15.4)	58 (71.0)	30	28	95.6	10 (7–14)
Mafeteng	37 (7.1)	20 (54.1)	13	7	77.0	14 (7–24)
Maseru	172 (33.2)	93 (54.1)	58	35	0	14 (7–25)
Mohale’s Hoek	53 (10.2)	34 (64.2)	26	8	123	8 (7–10)
Mokhotlong	4 (0.8)	2 (50.0)	1	1	291	6.5 (5–8)
Qacha’s Nek	32 (6.2)	18 (56.1)	11	7	224	10 (8–19)
Quthing	11 (2.1)	9 (82.0)	6	3	176	16 (9–23)
Thaba Tseka	13 (2.5)	10 (77.0)	8	2	170	60 (22–70)
Over-all						12 (7–19)

*Approximate distance, from patient district to the district where the MDR hospital is located.

Of the 314 patients seen at the MDR hospital, only 101 (32%) were further assessed for first-line drug susceptibility, while 81 (26%) had a specimen sent for second-line DST (Table 3). MDR-TB (resistance to isoniazid and rifampicin) was diagnosed in 57 (56.4%) and rifampicin mono-resistant TB in 34 (33.7%) patients. The proportion of patients with resistance to second-line anti-TB drugs cannot be accurately determine due to the high number for culture negative results (92.5%).

**Table 3:** Routinely collected phenotypic drug susceptibility testing results (Patients Diagnosed with RR-TB using GeneXpert).

Phenotypic DST	Resistance Profile*	Number	Percentage
First-line DST (N = 101)	Negative culture	7	6.9
	R	34	33.7
	H	2	2.0
	RH	46	45.5
	RHE	6	5.9
	RS	1	1.0
	RHS	2	2.0
	RHES	3	3.0
Second-line DST (N = 81)	Negative culture	75	92.5
	FQ	6	7.5

*R: rifampicin resistance, H: isoniazid resistance, RH: rifampicin and isoniazid resistance, RHE: rifampicin, isoniazid and ethambutol resistance, RS: rifampicin and streptomycin resistance, RHS: rifampicin, isoniazid and streptomycin resistance, RHES: rifampicin, isoniazid, ethambutol and streptomycin resistance, FQ: fluoroquinolone resistance. DST: drug susceptibility test.

### Treatment outcomes and associated factors

For treatment outcomes, we analyzed data on all patients who initiated treatment. Of the 314 patients, only 288 (91.7%) were eligible for treatment outcome analysis. We excluded four patients because of missing data on outcomes, four because they were still receiving treatment at the time of data collection and 18 were not evaluated. Overall the treatment success rate was 69.8% (201) and the death rate 28.8% (83). Only three patients were lost to follow up (1.0%), and one patient had treatment failure (0.4%).

We assessed factors such as age, gender, weight before treatment, HIV status, previous history of TB, and sputum smear result after one month of treatment for their association with the treatment outcomes. There was no evidence of association between HIV status and treatment outcome (Fisher’s exact p = 0.40), weight before treatment and treatment outcome (ANOVA F (2, 191) = 1.35, p = 0.3), history of previous TB and treatment outcome (Fisher’s exact p = 0.64) and, gender and treatment outcome (Fisher’s exact, p = 0.17). However, our study showed an association between sputum smear positivity after one month of treatment and treatment outcome as well as between age and treatment outcome. Patients with positive sputum smear after one month of treatment and those with no sputum smear result were more likely to die when compared to patients with a negative sputum smear after one month of treatment (Fisher’s exact, p < 0.001). Mean age between the three categories (treatment success, death and other poor outcomes) was different (ANOVA F (2,285) = 5.17, p = 0.0097) with older patients more likely to die compared to younger patients (Mean difference in age between those who were cured and those who died was 5.28 years, Bonferroni adjusted p = 0.007). There was no difference in age between patients with positive, negative, and unavailable sputum results at the end of month one (ANOVA F (2,283) = 2.34, p = 0.09). This indicates minimal risk of confounding between these two variables for their association with treatment outcomes.

The majority of patients were started on isoniazid, kanamycin, levofloxacin, prothionamide, cycloserine and para-amino salicylic acid as initial treatment regimen. We could not assess the association between the initial treatment regimen and treatment outcomes because some of the patients had their regimen adjusted after DST and/or medication side effects were reported. Less than 10%of patients had at least one of the new TB drugs (delaminid, bedaquiline) in their treatment regimen.

## Discussion

With this study we were able to determine the proportion of RR-TB cases linked to care, the turnaround time to treatment initiation, the implementation of confirmatory phenotypic DST and factors influencing treatment outcomes for patients in the Kingdom of Lesotho.

Our study is inclusive of patients coming from both urban and rural settings and in a context of centralized care where patients can only be initiated on treatment at one Hospital in Maseru, the capital of Lesotho. Only 60% of RR-TB cases could be linked to care, implying that the remaining 40% of cases were not on treatment, thereby possibly contributing to the transmission of drug resistant TB. Non-linked patients may have died prior to seeking care at the MDR hospital while others may have sought treatment outside of Lesotho.

For patients with record showing linkage to care and date of treatment initiation, the median time to treatment initiation was 12 days (IQR 7–19) despite the difficulty of transport in this mountainous terrain. This compares well with the median delay-time of 13 days (IQR 7–28) and 15 days (IQR 8–23) reported in Johannesburg and in the Eastern Cape in South Africa, respectively. In a Cape Town study, Cox *et al*. found a relatively shorter median time to treatment initiation of eight days^9–12^. In these studies, time to treatment initiation was measured from sputum submission at the laboratory while we measured it from the availability of a positive Xpert MTB/RIF assay result with rifampicin resistance. Given the Xpert MTB/RIF assay turnaround time, we assume that there is no significant difference between the two measurements.

Surprisingly, we noted a number of patients with negative treatment delay. These patients were on treatment for drug resistant TB prior to the diagnosis of RR-TB at the GeneXpert facility^13^. The Lesotho Tuberculosis guidelines need to emphasize the value of using the Xpert MTB/RIF assay to diagnose RR-TB in patients already on treatment, as the current WHO guidelines do not recommend the use of the Xpert MTB/RIF assay to monitor patients on anti-TB treatment^13^.

Only 32% of patients on standardized drug resistant treatment had a confirmatory phenotypic DST despite this being done at the Lesotho National Tuberculosis Laboratory. This implies that the vast majority of patients with RR-TB were treated without the knowledge of other potential resistances, which could explain the high death rate. The high proportion of rifampicin mono-resistance is similar to what has been reported in South Africa^14,15^ and has been linked to acquisition through malabsorption associated with HIV co-infection^16–18^. However, we cannot exclude the possibility of ongoing transmission, as approximately 50% of patients diagnosed with RR-TB were new cases. Unfortunately, the extent of second-line resistance in RR-TB cannot be assessed from this study, as only 7% of DSTs requested gave an actionable result by demonstrating the presence of resistance to a fluoroquinolone. The yield of actionable results may be improved using a molecular DST method such as the GenoType MTBDR*sl* assay (second-line LPA) endorsed by the WHO^19^.

Despite limited knowledge of the resistance profiles of the strains circulating in Lesotho, treatment success was observed in 69.8% of patients, death in 28.8%, loss to follow up in 1.0% and treatment failure in 0.4%. Only the sputum smear results at the end of the first month of treatment were strongly associated with poor treatment outcomes. Treatment success and death rates observed in our study are similar to results reported by the Lesotho National Tuberculosis program, respectively 67% and 28%^7^. The treatment success rate is greater than the global treatment success rate of 55% reported by the 2018 Tuberculosis report and greater than the 39% reported by a study in Johannesburg, South Africa but lower compared to the global target. The death rate was higher than that reported globally (15%) and in South Africa (14%) but similar to death rates observed in Mozambique (26%)^1^.

Data for this study was collected from routine records from the GeneXpert facilities and MDR hospital. Since routine records are not intended for research, they are not recorded systematically. This may have led to under- and/or overestimation of some of our findings. Our estimate of linkage to care may be an under-representation given that the linkage to care was not physical but based on information recorded at the GeneXpert facilities and the MDR Hospital. Patients’ names in the registers we used could have been recorded differently between the GeneXpert facility and the MDR hospital, although we tried to overcome this by checking different spelling, date of birth, sex and referring facility. The high rates of treatment success may be due survival effect, many patients may have died before linkage to treatment and were therefor not included in our analysis.

In summary, new strategies need to be implemented to increase the proportion of RR-TB cases linked to care. We suggest improving communication between the referring GeneXpert facility and the MDR-TB hospital to ensure follow-up of patient who have not initiated treatment, provision of transport between the referring facility and the MDR-TB hospital and capture of patient contact details to enable follow-up. Such strategies may also impact on the time to MDR-TB treatment initiation to meet the Lesotho National Tuberculosis program guidelines. To further improve treatment outcomes, DST should be implemented for all patients diagnosed with RR-TB. This will also limit the selection of *M. tuberculosis* strains with drug resistance beyond MDR.

## Methods

### Study design

A historical cohort study of patients diagnosed with RR-TB using the Xpert MTB/RIF assay from the 1^st^ of January 2014 to the 31^st^ of December 2016 was conducted. All patients aged 18 years and above, diagnosed with RR-TB, were included.

### Ethical approval

This study was approved by the Health Research Ethics Committee at Stellenbosch University and the Lesotho Ministry of Health Research committee who granted permission to perform a retrospective folder review without informed consent as there would be no contact with the patients. The ethical approval from the Lesotho Ministry of Health was sufficient for data collection in Lesotho’s public hospitals after an arrangement with either the Hospital Medical Superintendent or the Laboratory Manager. For Christian Health Association of Lesotho (CHAL) hospitals, we obtained additional authorizations from CHAL’s executive director and from each member of CHAL’s hospital management team. For the Lesotho MDR hospital, we obtained authorization from The Lesotho Partners in Health (PIH) that run the hospital. This research was conducted in accordance with guidelines and regulations as set out by the ethical approval committees of the respective institutions.

### Study sites

GeneXpert facilities at hospitals in 10 districts in Lesotho [Maseru District (Queen Elisabeth II Hospital, Teba Clinic, National Tuberculosis Reference Laboratory, Scott Hospital, Saint Joseph Hospital, Lesotho MDR Hospital), Mafeteng District (Mafeteng Hospital), Mohale’s Hoek District (Ntsekhe Hospital), Quthing District (Quthing Hospital), Qacha’s Nek District (Machabeng Hospital, Tebellong Hospital), Berea District (Berea Hospital, Maluti Adventist Hospital), Leribe District (Teba clinic, Motebang Hospital), Butha Buthe District (Butha Buthe Hospital, Seboche Hospital), Thaba Tseka District (Saint James Hospital and Paray Hospital), Mokhotlong District (Mokhotlong Hospital)] were included for Xpert MTB/RIF data collection (Fig. 2). We did not collect data from GeneXpert facilities located at Mamohau where we did not get the approval from the hospital management and at the Lesotho MDR Hospital due to time constraints.

### Sampling method

Data were collected in two phases; the first phase consisted of diagnostic data collection done at GeneXpert facilities and the second phase consisted of clinical data collection done at the hospital where patients with drug resistant TB were treated. Most of the GeneXpert facilities used an electronic data storage system (DISA) with the exception of three centers (Queen Elisabeth II Hospital, Teba Clinic Maseru and Teba Clinic Leribe) that used manual registers.

For GeneXpert facilities using DISA, we searched for all patients with an Xpert MTB/RIF assay result and later filtered for patients in whom RR-TB was diagnosed. Patients with RR-TB were consecutively included in our study if they met all study entry criteria (age 18 years and above, diagnosis of pulmonary RR-TB using Xpert MTB/RIF between January 2014 and December 2016). For GeneXpert facilities using manual registers, patients were included in the study after they were identified as having RR-TB in the register.

Patients diagnosed with RR-TB at the respective GeneXpert facilities were then searched for in the Lesotho MDR Hospital (Botsabelo) records as this is the only hospital in the country accredited to provide comprehensive Tuberculosis drug resistant treatment. Patients were matched using their full name as recorded at the GeneXpert facilities. We checked on different name’s spelling, date of birth, sex and referring facility to confirm if the patient matched was the correct patient. All patients identified at the GeneXpert facilities had a minimum follow up time of 24 months to access treatment. For patients who were successfully matched, folders were reviewed for data collection.

For patients that were matched at the hospital, we collected the following information: arrival date at hospital, initiation date of treatment, physical address, occupation, weight before treatment initiation and after treatment completion, HIV status, history of previous TB and method of diagnosis, treatment outcome of previous TB, treatment regimen of current episode of TB, sputum smear results from month 1 to month 24, first-line drug susceptibility results, second-line drug susceptibility results, treatment outcome. We were, however, unable to collect information on patients’ concurrent morbidities, treatment side effects, viral load (if HIV positive) and CD4 counts. This information could have provided reasons for a patient failing to initiate treatment or their response to treatment.

We defined treatment delay as the interval between the date of availability of a positive Xpert MTB/RIF result (with rifampicin resistance) and the date of initiation on MDR-TB treatment. Patients with positive treatment delay (those initially diagnosed at the visited GeneXpert facility and initiated on treatment afterward) and negative delay (patients who were already on treatment at the time of diagnosis at the GeneXpert facility) were included in the analysis. Treatment outcomes were recorded as per the WHO definitions (cure, failure, lost to follow up, completed, died, not evaluated), treatment success rate is given by the sum of cure and completed^20^. Due to low numbers of observations in some categories (one for treatment failure and two for lost to follow up), we reduced the number of categories to three (treatment success, death and other poor outcomes). Distance to the MDR hospital was calculated using google map and represent the distance from the district where the patient was diagnosed with RR-TB to Maseru where the MDR Hospital is located.

### Statistical analysis

Data collected were analyzed using Stata 14.0. Demographics of study participants were presented using frequency tables, mean, standard deviation and 95% confidence interval (CI) for normally distributed data or median and interquartile range for skewed data. Normally distributed data were analyzed using one-way ANOVA with post hoc Bonferroni tests. Data that were not normally distributed were analyzed using Wilcoxon Rank Sum test (Mann-Whitney), Kruskal Wallis tests and Spearman’s rank correlation. Categorical data were analyzed using Chi squared and Fisher exact tests. A p value < 0.05 was considered as statically significant.

## Supplementary information

Supplementary Dataset 1.

## Data Availability

All data generated or analyzed during this study are included in Supplemental Data.
